# IPD-IMGT/HLA Database

**DOI:** 10.1093/nar/gkz950

**Published:** 2019-10-31

**Authors:** James Robinson, Dominic J Barker, Xenia Georgiou, Michael A Cooper, Paul Flicek, Steven G E Marsh

**Affiliations:** 1 Anthony Nolan Research Institute, London, UK; 2 UCL Cancer Institute, University College London (UCL), London, UK; 3 European Molecular Biology Laboratory, European Bioinformatics Institute, Cambridge, UK

## Abstract

The IPD-IMGT/HLA Database, http://www.ebi.ac.uk/ipd/imgt/hla/, currently contains over 25 000 allele sequence for 45 genes, which are located within the Major Histocompatibility Complex (MHC) of the human genome. This region is the most polymorphic region of the human genome, and the levels of polymorphism seen exceed most other genes. Some of the genes have several thousand variants and are now termed hyperpolymorphic, rather than just simply polymorphic. The IPD-IMGT/HLA Database has provided a stable, highly accessible, user-friendly repository for this information, providing the scientific and medical community access to the many variant sequences of this gene system, that are critical for the successful outcome of transplantation. The number of currently known variants, and dramatic increase in the number of new variants being identified has necessitated a dedicated resource with custom tools for curation and publication. The challenge for the database is to continue to provide a highly curated database of sequence variants, while supporting the increased number of submissions and complexity of sequences. In order to do this, traditional methods of accessing and presenting data will be challenged, and new methods will need to be utilized to keep pace with new discoveries.

## INTRODUCTION

Over twenty years ago, the HLA Informatics group of the Anthony Nolan Research Institute, released the IMGT/HLA Database (1). Since this time, this online resource has acted as the repository for the innumerable variant sequences of HLA alleles named by the WHO Nomenclature Committee for Factors of the HLA System (1–10). For over 50 years (11,12), the nomenclature committee have been responsible for naming the genes and allelic variants of the HLA genes, found within the human Major Histocompatibility Complex (MHC) (13–26). In 2003, the IMGT/HLA Database was incorporated into the Immuno-Polymorphism Database (IPD) project alongside variant databases covering Killer-cell Immunoglobulin-like Receptors (KIR) and sequences from the non-human Major Histocompatibility Complex (MHC). The IPD-IMGT/HLA Database has provided a stable, highly accessible, user-friendly repository for this information.

**Figure 1.**
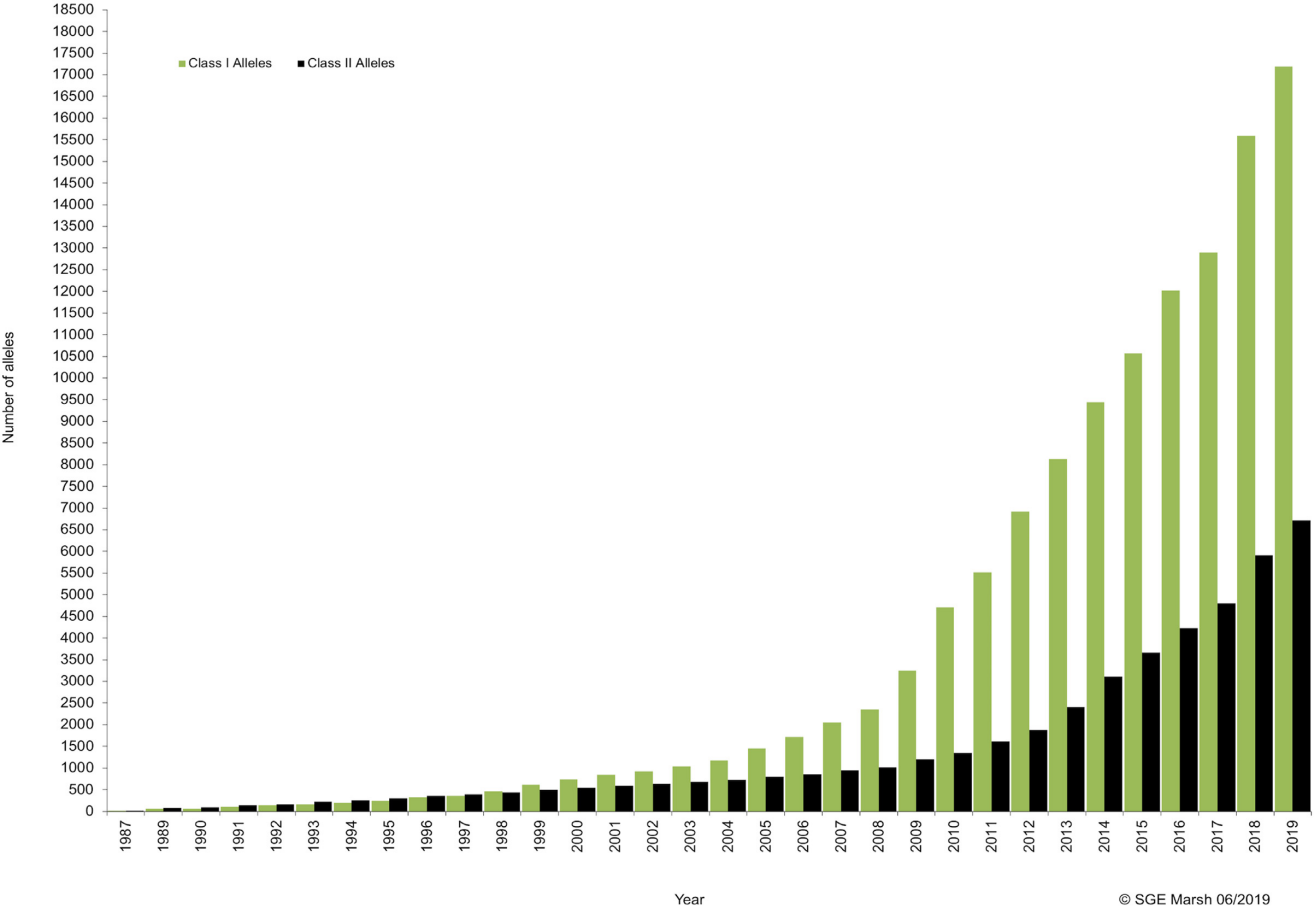
The number of HLA alleles named by the WHO Nomenclature Committee for Factors of the HLA System and included in the IPD-IMGT/HLA Database, show a continuous increase in new alleles identified both for class I and class II over the past 30 years. The rate of discovery of new alleles continues to increase with the use of next-generation sequencing technologies, with more new alleles named in the first three months of 2019, than were named in the last century.

The genes included in the IPD-IMGT/HLA Database lie within the MHC and the extended MHC region. The MHC is a region in the genome of all jawed vertebrates, and encodes core components of the immune system (27). In humans, it is referred to as HLA. The extended MHC region covers a number of other genes in close proximity, including HFE. The hallmarks of the MHC are that it contains highly polymorphic genes that encode diverse antigen-presenting molecules (28). Their function is to generate proteins, which bind to peptides generated from infecting pathogens and present them to the immune system. The HLA genes are stable in structure and organisation, and importantly, co-dominant in expression. The HLA complex, around 4 million bases in length, is located on chromosome 6, 6p21.3 (29). The HLA genes with this region are known to be highly variable, in fact the MHC region is the most polymorphic region of the human genome and the level of diversity seen has been described as ‘hyperpolymorphic’ rather than simply polymorphic (30). Within the HLA field the term ‘allele’ refers to the combination of point mutations, insertions and deletions that are seen in a single phased sequence for any given gene. Each allele can therefore be made up of multiple variant positions when compared to a single reference sequence. To this end the IPD-IMGT/HLA Database, as of August 2019, contains 24,093 HLA and related alleles, comprised of over 362,709 distinct nucleotide variants compared to the reference sequence, at 86,902 of the 234,539 curated positions, see Figure 1 and Table 1.

**Table 1.** Levels of variation in HLA'Can the table also be better aligned, as the columns and headers are not in sync with each other.

Gene	Length (bp)	Distinct variant bases	Distinct variant positions	Alleles
*HLA class I genes*				
HLA-A	3868	6736	1716	5266
HLA-B	4235	6863	1535	6537
HLA-C	4503	7018	1540	5140
HLA-E	3895	3993	98	43
HLA-F	3567	3651	80	44
HLA-G	3151	3250	98	69
*HLA class II genes*				
HLA-DRA	5721	5825	103	7
HLA-DRB1	18522	30535	10415	2581
HLA-DRB3	13679	27135	13060	305
HLA-DRB4	15492	40757	15074	153
HLA-DRB5	13508	26452	12835	122
HLA-DQA1	6776	8365	1428	183
HLA-DQB1	7780	11541	3136	1718
HLA-DPA1	9842	10520	656	132
HLA-DPB1	11620	12950	1058	1449
HLA-DMA	5023	5037	14	7
HLA-DMB	6752	6771	18	13
HLA-DOA	3663	3690	27	12
HLA-DOB	4798	4822	24	13
*HLA class I pseudogenes*				
HLA-H	3688	3846	155	25
HLA-J	3565	3629	64	9
HLA-K	3578	3710	128	6
HLA-L	3787	3905	118	5
HLA-P	2956	3010	54	5
HLA-T	2496	2575	79	8
HLA-V	1914	1966	52	3
HLA-W	2987	3115	125	11
HLA-Y	3107	4192	1083	3
*HLA class II pseudogenes*				
HLA-DRB2	1086	1419	333	2
HLA-DRB6	1086	1167	81	4
HLA-DRB7	1086	1149	63	3
HLA-DRB8	1086	1108	22	2
HLA-DRB9	1086	1162	75	7
HLA-DPA2	6810	6929	119	5
HLA-DPB2	17911	18574	660	6
*Other genes within the MHC*				
HFE	7977	7994	16	6
MICA	12866	13261	391	109
MICB	12901	24856	11773	47
TAP1	9294	9322	28	12
TAP2	10642	19279	8568	12

## TOOLS AVAILABLE AT IPD-IMGT/HLA

The IPD-IMGT/HLA Database provides a large number of tools for the analysis of HLA sequences. These tools are either custom written for the database or data libraries have been incorporated into the existing tools available from the European Molecular Biology Laboratory's European Bioinformatics Institute (EMBL-EBI) website.

- Sequence alignments: access to an alignment tool, which filter pre-generated alignments to the user's specification. Provides alignments at the protein, cDNA and gDNA level. The original alignment tools were developed to view a smaller number of alleles over a smaller sequence range. The increased number of new alleles and sequence coverage has necessitated the development of new faster, more interactive versions of this tool to better aid the user in selecting and viewing the alignments, these are currently been utilised by the IPD-MHC project (http://www.ebi.ac.uk/ipd/mhc/align.php), and will also be used for the IPD-IMGT/HLA
- Allele queries: access to detailed information on any HLA Allele, including information on the ethnic origin of the source material, database cross-references and seminal publications. This information is also available through integration with the EB-eye Search Tool (31).
- Sequence search tools: integration into EMBL-EBI’s suite of search tools like EB-eye, and the inclusion of IMGT/HLA datasets as searchable libraries in the FASTA and BLAST tools provided by EMBL-EBI (32,33).
- Cell Queries: a detailed and searchable database of all the source material characterised in the submissions.
- Downloads: access to either an FTP directory located on the EMBL-EBI server or a GitHub repository containing all the data from the current and previous releases in a variety of commonly used formats like FASTA, MSF and PIR.

## DATA SOURCES

**Figure 2.**
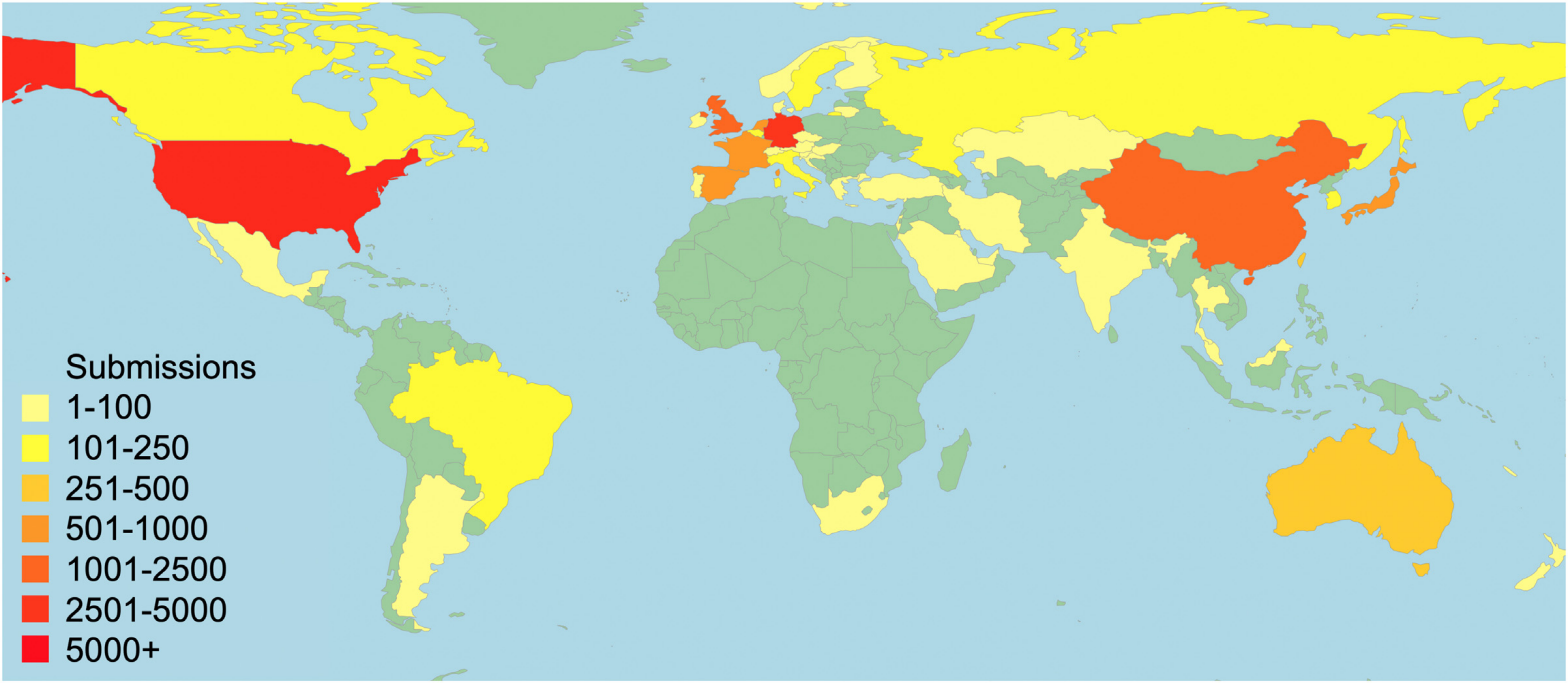
The IPD-IMGT/HLA Database receives submissions from over 46 countries. The figure shows the location of submitters to the database and the volume received from each country. It should be noted that the source material may be derived from a number of additional countries, and only the location of the submitting laboratory is shown on the map.

**Figure 3.**
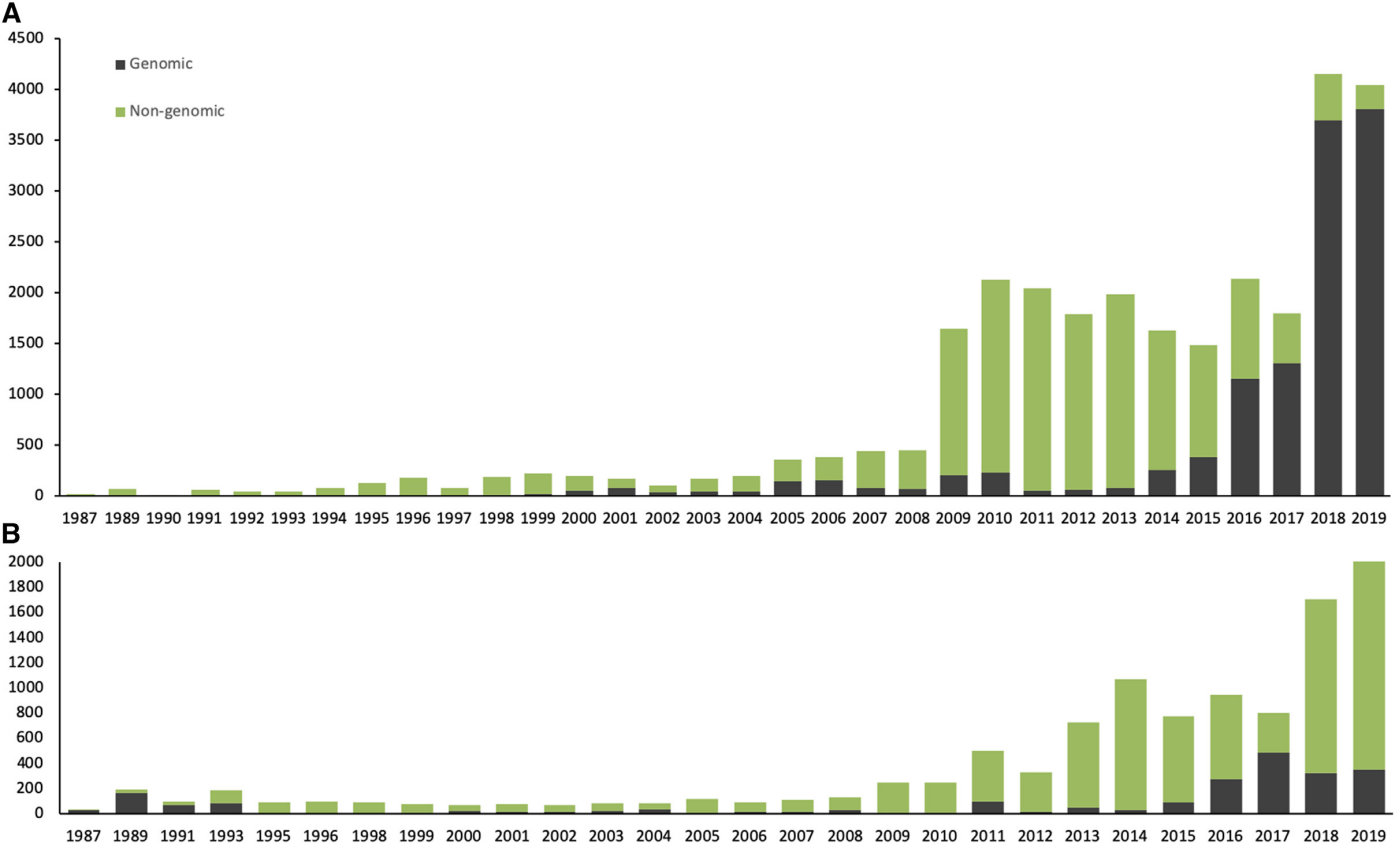
The IPD-IMGT/HLA Database has received over 53,000 submissions since its launch in 1998. This figure shows the number of submissions annually as well as a breakdown of how the type of submissions has changed with the incorporation of new technologies. Panel **A** represents HLA class I submissions and panel **B** represents HLA class II submissions. The advent of technologies capable of routinely sequencing the length of the class I genes, ∼3500 bp, has led to an increase in the number of full-length (5′ UTR to 3′ UTR) submissions, compared to partial submissions covering just exons 2–3, that were previously the norm. The HLA class II introns are substantially longer, and whilst more genomic sequences have been received, the shorter partial sequences, covering just exon 2, still form the majority of submissions.

The IPD-IMGT/HLA Database receives submissions from laboratories in over 46 countries and active website uses from users in over 150 countries, see Figure 2. These submissions are curated and analysed, and if they meet the strict requirements an official allele designation is assigned. The IPD-IMGT/HLA Database is the official repository for the WHO Nomenclature Committee for Factors of the HLA System, and this is the only way of receiving an official allele designation for a sequence. The sequence is then incorporated into the next three-monthly release of the database. Since its release in December 1998 the database has received over 53,410 submissions, from over 1,079 submitters, see Figure 3. These submissions come from a variety of sources; the majority are from routine HLA Typing laboratories or companies performing contract HLA typing often for large bone marrow donor registries. Other submissions are from large-scale genome typing projects. All submissions must meet strict acceptance criteria before the sequence receives an official designation, around 4% of the submissions received fail to meet these criteria and are rejected. In addition, all the submissions received by the IPD-IMGT/HLA database are also available from the member databases of the International Nucleotide Sequence Database Collaboration (INSDC) (34–37). The EMBL–European Nucleotide Archive entries also contain database cross-references to the IPD-IMGT/HLA Database entries.

Submissions are processed on a monthly basis and every 3 months, the publicly available copy of the database is updated, along with all tools and data repositories. As part of the release process, key files included in the release are sent to a third-party for quality review, and to identify potential errors in allele assignment or formatting errors in various file formats. Many of these formats were originally designed when the complexity and volume of data was much lower. With the increase in sequence complexity every effort is made to ensure sequences with complex indels or splice variants are accurately included in these formats. The inclusion of new and complex features is not always straightforward, and despite these checks, sometimes errors may be found in file formats. To help record, track and fix these, the GitHub repository provides an issue tracker that allows users to report these potential issues, which can be investigated and acted upon by the team where necessary. The existing formats are all reviewed regularly to ensure that they are still fit for purposes and suitable for the increased volume and complexity of the data. The processes for the annotation and subsequent publication of the data in multiple formats or via the website are also under constant review. At the current time the code used for the management of the database and publication of the numerous file formats is not available as part of the GitHub repository.

## NGS AND CHANGES IN SEQUENCING TECHNOLOGIES

One of the major challenges facing the IPD-IMGT/HLA Database over more recent years has been the developments in sequencing technologies (38). Over the past 30 years the laboratory techniques used to identify HLA alleles have improved and this has led to wider availability to allow for testing and analysis of samples. Prior to 1998 and the initial release of the IPD-IMGT/HLA Database the majority of sequences were identified with serological methods, being defined by anomalous reaction patterns, and consequently only a limited number of alleles were identified and sequenced. Over the past decade high resolution high-volume sequence-based HLA typing has become readily available to many laboratories, whether using Sanger sequencing (39), or more recently both short-read Next Generation Sequencing (NGS) (40) methods or long-read Next or Third Generation Sequencing (TGS) methods (41). The more recent developments in sequencing have allowed for increased accuracy and coverage of full-gene sequences. As a result of these developments over the previous 4 years the IPD-IMGT/HLA Database is now receiving more full-length gene sequences for class I than partial sequences covering just exons 2 and 3. The minimum requirement for the submission of a class I sequence currently remains exons 2 and 3, and this is the most characterized region and most submitted region as it encodes the peptide binding domain of the HLA molecule.

A key aim is to expand the coverage of the region, to cover the remaining pseudogenes. These are often duplicated fragments of the expressed genes, and whilst their functional role can be debated, the inclusion of their sequences aid in their identification in NGS typing, where they can be mistaken for expressed alleles.

## SEQUENCE VARIATION

**Figure 4.**
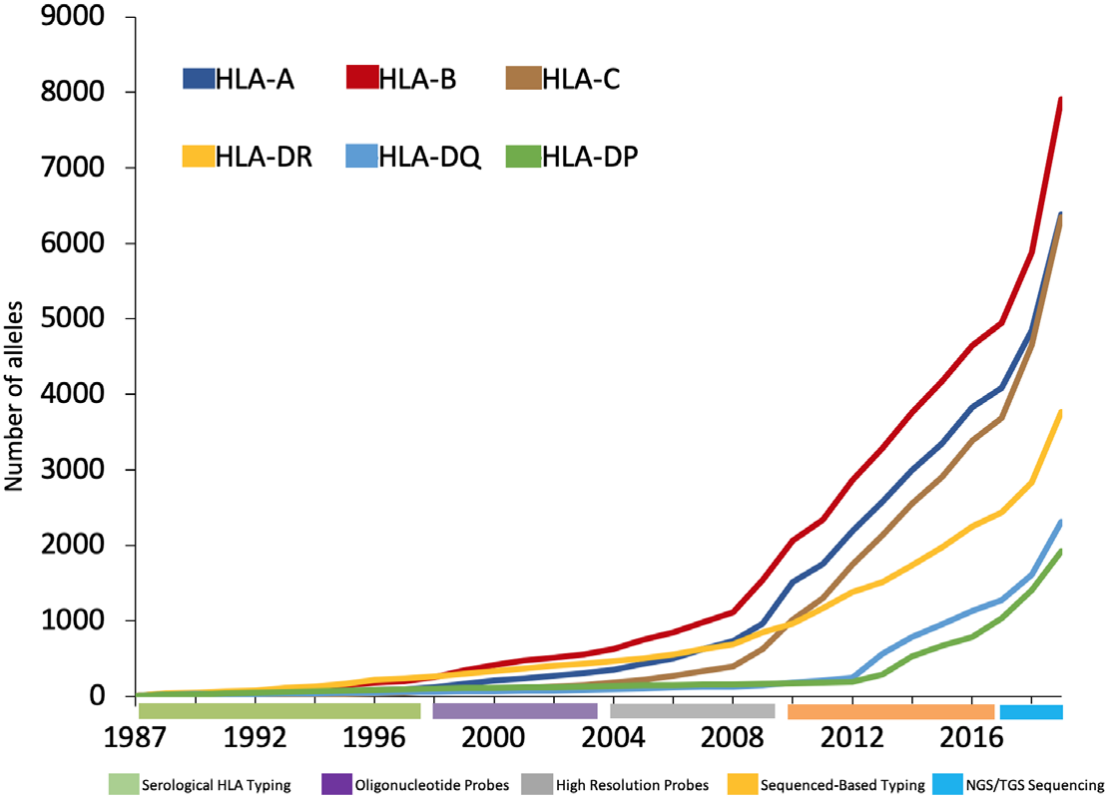
Growth in the number of recognized class I and class II alleles across different typing and sequencing technologies, adapted from Robinson *et al.* (30).

**Figure 5.**
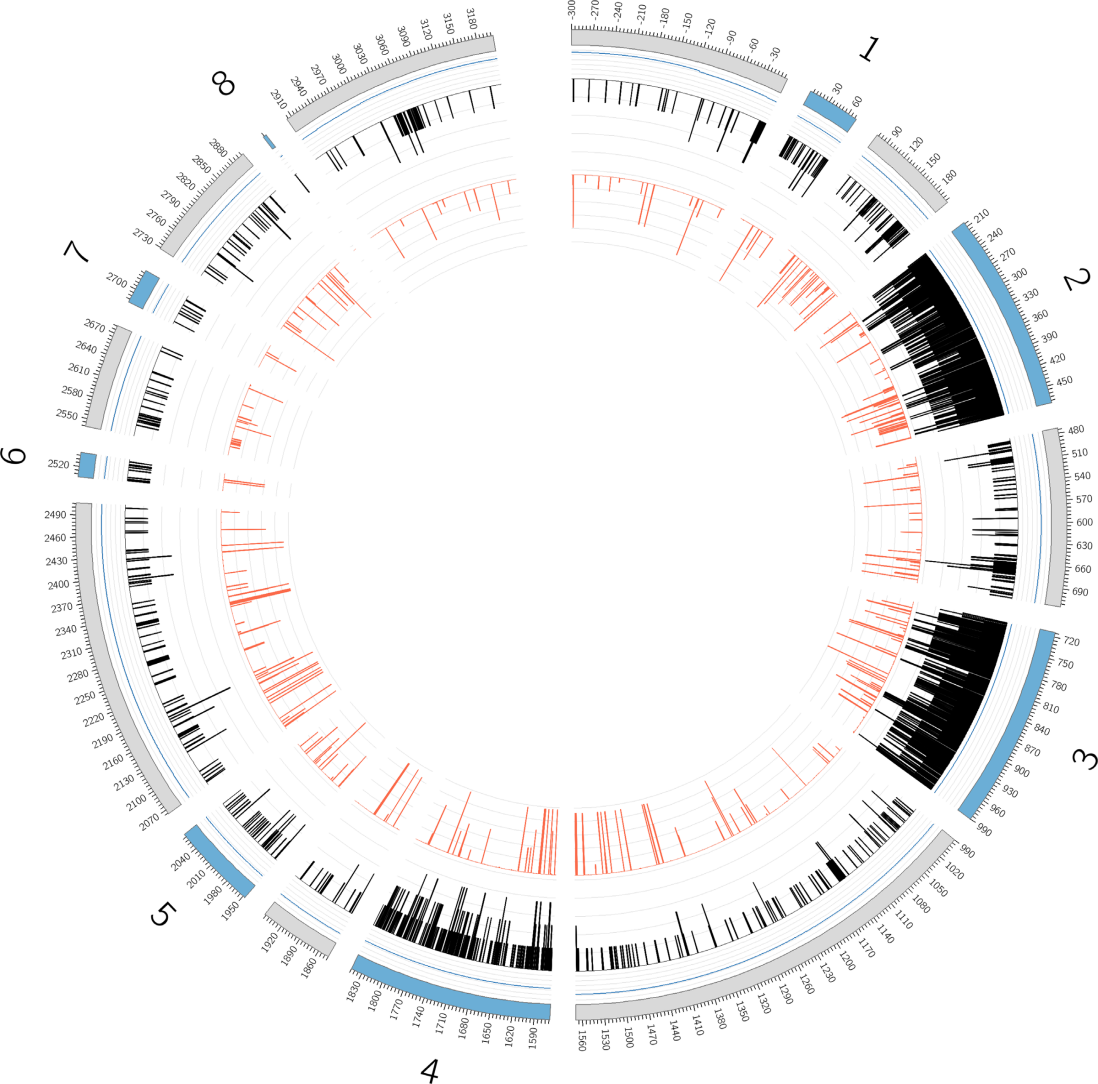
A graphical representation of the variation seen in the HLA-A sequences. Moving from the perimeter towards the centre of the diagram, the outer ring represents the different regions, with the exons filled in blue and numbered, the gDNA positions are also shown. The next layer represents the percentage of alleles with sequence in the database, the further towards the centre, the higher the percentage of alleles with sequence, note exons 2 and 3 where this sequence is mandatory for acceptance in the database. The penultimate inner ring represents the numbers of bases, (A, C, G, T or an indel) seen at each position with the baseline representing a monomorphic position. The final inner ring shows in red, the frequency of the second most common base at each position. The diagram can therefore be seen to show that whilst variation is highest in exons 2 and 3, it is not limited to these regions and there are clear regions of conserved variation throughout the gene.

With this increase in the volume of submissions, has also come an increase in the complexity of the submissions. Figure 4 shows how the rate of allele discovery has changed with time, to reflect different techniques. Within recent years, the advent of NGS and TGS technologies has also led to longer sequences being submitted. Whilst the IPD-IMGT/HLA Database has acted as a repository for cataloguing the variation in the HLA genes, through the work of the WHO Nomenclature Committee for Factors of the HLA system, the project does not aim to quantify or qualify the variation. With the rapidly increasing numbers of submissions and subsequently assigned alleles, it is imperative that the database continues to catalogue these variants but also facilitates research into how this unparalleled level of variation is generated, maintained and continues to grow. A number of studies of the polymorphisms within HLA have focussed on the MHC region as a whole, or looked at the evolution of this complex region (28,42–51). Our recent studies (30) have shown that the levels of polymorphisms seen with HLA class I genes are predominantly down to point mutations. These point mutations are commonly seen accumulating around common frequently seen alleles. The other source of variation is through recombination of these more commonly seen variants. Under-pinning this are a number of core alleles that form a backbone to variants seen and shuffling of motifs from these core alleles through both intra- and inter-genic recombination has given rise to many of the serological groups. The increased numbers of alleles identified over recent years, is predominately due to single point mutations, particularly as these variants are more easily identified using sequencing methodologies. Previous techniques based on probe patterns often overlooked variations in intervening regions between probe sites. The latest visualizations of the data developed using Circos (52), see Figure 5, show that the variation is also not limited to the peptide recognition domain, which has been the focus of the majority of sequences submitted to the database, as previously imagined, and as such sequencing of the full gene, will be paramount to understanding sequence variation in HLA. The IPD-IMGT/HLA Database will need to provide tools for collection, analysis and visualization of the full gene sequence.

## DISCUSSION

The challenge for the IPD-IMGT/HLA Database is to continue to provide a highly curated database of sequence variants, while supporting the increased number of submissions and complexity of sequences. In order to do this, traditional methods of accessing and presenting data will be challenged, and new methods utilizing new computing technologies will need to be utilized to keep pace with a shifting user focus. Recent studies (30, 51) have suggested that the potential number of HLA alleles could be as high as 2–3 million for HLA-A, -B and -C. This prediction is based on analysis of the exons 2 and 3 sequences and does not include measures of variation outside of these regions. The changing dynamic in the volume and complexity of the sequences received in the last few years, suggests that if variation is considered outside of the peptide binding domain then even several million variants per gene may be an underestimate. Despite these estimates, it is clear that whilst the human population has a small number of common HLA class I alleles that are present at appreciable frequency in different populations, the overwhelming majority of HLA class I alleles are very rare and highly localized in their distribution. The challenge for the database is providing not only an infrastructure capable of handling the influx of data, but also through the HLA nomenclature a methodology for naming the variants identified. This will also present the database with challenges in disseminating the information to both the more traditional users of HLA data in clinical laboratories to a new wave of genomics studies based on large data sets of variant sequences, and individual variant positions.

## DATA AVAILABILITY

The IPD-IMGT/HLA Database can be accessed at https://www.ebi.ac.uk/ipd/imgt/hla/.

The IPD-IMGT/HLA Database provides an FTP site for the retrieval of sequences in a number of pre-formatted files. The sequences are provided as FASTA, PIR and MSF formats, as well as an archive of the sequence alignments and a flat file formatted copy of the database. The FTP directory is available at the following address: ftp://ftp.ebi.ac.uk/pub/databases/ipd/imgt/hla/.

Both current and previous releases are archived in a Git repository and available at: https://github.com/ANHIG/IMGTHLA . This repository contains a branch for each database release and a Latest branch which contains the most recent files as well as all compressed archives.

For more information about the database, queries or to subscribe to the IPD mailing lists please contact hla@alleles.org.
